# Strengthening research ethics governance and regulatory oversight in Central America and the Dominican Republic in response to the COVID-19 pandemic: a qualitative study

**DOI:** 10.1186/s12961-022-00933-z

**Published:** 2022-12-23

**Authors:** Julio Arturo Canario Guzmán, Jessie Orlich, Renata Mendizábal-Cabrera, Argentina Ying, Claude Vergès, Eleonora Espinoza, Mario Soriano, Elsy Cárcamo, Briana Beltrán, Eddys Rafael Mendoza Marrero, Reyna Sepulveda, Claudia Nieto Anderson, Nicole Feune de Colombi, Roxana Lescano, Eddy Pérez-Then, Trudie Lang, Jackeline Alger

**Affiliations:** 1 Fundación Etikos, Inc., Santo Domingo, Dominican Republic; 2 Centro Nacional de Investigaciones en Salud Materno Infantil Dr. Hugo Mendoza (CENISMI), Santo Domingo, Dominican Republic; 3 Instituto Costarricense de Investigaciones Clínicas, San Jose, Costa Rica; 4 Centro de Estudios en Salud, Universidad del Valle de Guatemala, Guatemala, Guatemala; 5 Universidad de Panamá, Panama, Panama; 6 Facultad de Ciencias Médicas, Universidad Nacional Autónoma de Honduras (UNAH), Tegucigalpa, Honduras; 7 Instituto de Enfermedades Infecciosas y Parasitología Antonio Vidal, Tegucigalpa, Honduras; 8 Comisión Nacional Ética de Investigación en Salud, San Salvador, El Salvador; 9 Centro Nacional de Educación Médica Continua (CENEMEC), Colegio Médico de Honduras, Tegucigalpa, Honduras; 10 Nuffield Department of Medicine, University of Oxford, Oxford, United Kingdom; 11 Asociación para el Empleo de Animales en Investigación y Docencia, ASOPEBAID, Lima, Peru; 12 Two Oceans in Health, Santo Domingo, Dominican Republic

**Keywords:** Central America, Collaboration, Community engagement, COVID-19, Dominican Republic, Governance, Health emergencies, Regulations, Research ethics

## Abstract

**Background:**

Good governance and regulatory supervision are required to conduct research in an international public health emergency context and to ensure compliance with ethical standards. The “Strengthening research ethics governance and regulatory oversight in Central America and the Dominican Republic in response to the COVID-19 pandemic” study is a regional effort in which research ethics stakeholders participated in addressing research ethics governance and preparedness response challenges to the COVID-19 pandemic in Central America and the Dominican Republic.

**Methods:**

A qualitative action research study was conducted following a participatory approach. Research ethics stakeholders in Central America and the Dominican Republic were mapped; a regional webinar and three virtual workshops were conducted discussing research ethics governance, ethics review and collaborative research practice during the pandemic. A roundtable session presented results and obtained feedback on a draft of a policy to strengthen regional research ethics governance.

**Results:**

Countries across Central America and the Dominican Republic are at different stages in their development of research ethics systems. Countries with more established systems before COVID-19 were better organized and prepared to respond. This finding argues against improvisation and supports further work on strengthening governance of research ethics systems. Community engagement in research ethics public policy-making is practically absent in the region. Research and research ethics collaboration schemes are lacking amongst the countries; however, there are incipient initiatives in the region, such as the Central America and Caribbean Network of Research Ethics Committees. A policy brief with recommendations on how to advance towards strengthening the governance of research ethics systems was prepared and submitted to the Central American Integration System for analysis and possible approval.

**Conclusion:**

National research ethics systems in Central America and the Dominican Republic were unprepared to respond to the COVID-19 pandemic with respect to research oversight and effective collaboration. In most cases, national research ethics systems were found to be weak, and regional research collaboration was practically absent. To promote collaboration, a joint strategy needs to be developed with a regional vision towards sharing knowledge and best practices.

**Supplementary Information:**

The online version contains supplementary material available at 10.1186/s12961-022-00933-z.

## Background

### Strengthening governance of health research systems

A coordinated worldwide research response is needed to fight the COVID-19 pandemic. To accomplish this, ethical and regulatory challenges must be overcome [1, 2]. Governance is a core function of health research systems. According to WHO, research governance must be strengthened in ways that allow for effective, efficient and ethical collaboration among multiple stakeholders [1].

### Defining research ethics systems and their governance

Hyder et al. [3] describe research ethics systems as “a component of the stewardship function of health research systems”. Since governance has been identified as a core function of health systems [4] and national health research systems (NHRS) [5], research ethics itself, as a system, requires a governance function to secure achievement of its objectives.

Several authors have addressed the topic of governance and frameworks for research ethics in times of global health emergencies [3–11]. The Pan American Health Organization (PAHO) developed a systemic approach with two strategic lines of action, objectives and indicators for strengthening the national research ethics systems (NRES) and emergency preparedness [12]. The governance function of such a NRES has not been fully described and needs to be further operationalized as general orientation for those aiming to implement effective research ethics systems.

In this paper, we refer to research ethics systems as the actors, institutions and activities whose primary purpose in research is to ensure ethical standards and procedures in the conduct of human research. The governance function of a research ethics system *implies the ability to formulate strategic policy direction, ensure good regulation, set and monitor ethical standards, and ensure accountability and transparency*. Hence, such research ethics governance includes mechanisms to ensure the functioning of the system to achieve compliance. The Virtual Health Library (VHL) defines health sector stewardship/governance as the “participation of stakeholders who are concerned with the definition and implementation of policies, programmes and practices that promote equitable and sustainable health systems” [13]. In this definition of governance, we underscore the participatory and democratic nature that concerns experts and the public alike. In this effort, we embraced this “participatory” view of governance rather than the mere exercise of authority, control, administration and government power to design, formulate and implement policies [14].

### Health research and research ethics: regional context

Despite advances in NHRS in the region, fragmentation and lack of coordination have been noted previously [15, 16]. In Latin America, research priorities for the COVID-19 pandemic were identified with participants from various countries [17]; however, the status of adoption of these recommendations by national authorities remains unknown. Thus, by early 2021, issues surrounding research ethics governance and oversight during the COVID-19 pandemic, specifically on Guideline 20 related to ethics review procedures in emergency situations as identified in the International Ethical Guidelines for Health-related Research Involving Humans [18], had not yet been assessed, and was an evident topic to be addressed in this region and globally. Additionally, we considered that research stakeholders’ insight regarding policies and practices of the research ethics system as a response to the pandemic, community engagement in health research and scientific collaboration were relevant topics to better understand the dynamics of research ethics systems in these countries.

To answer these questions, an international collaborative research study titled “Strengthening research ethics governance and regulatory oversight in Central America and the Dominican Republic in response to the COVID-19 pandemic” (the GoEtiCA study) was proposed. The goal was to determine the status of research ethics systems in the Central American and Dominican Republic region (CA-DR), specifically in the context of the COVID-19 pandemic, and to identify scientific and community collaborations that emerged in response to this emergency. These goals were considered a first step to promote the development, formalization and adoption of a set of recommendations for improving the research ethics governance with an emphasis on public health emergencies (PHEs) in Central America and the Dominican Republic.

## Methods

This is an action research study, based on a qualitative participatory approach [19]. An interdisciplinary and international research team was formed, including at least one research ethics expert representing each of the countries involved in the study. An international advisory group of experienced and recognized professionals in the fields of research ethics and global health was also established. Members of the advisory committee for the project came from Peru, Argentina, the Dominican Republic and the United Kingdom.

**Fig. 1:**
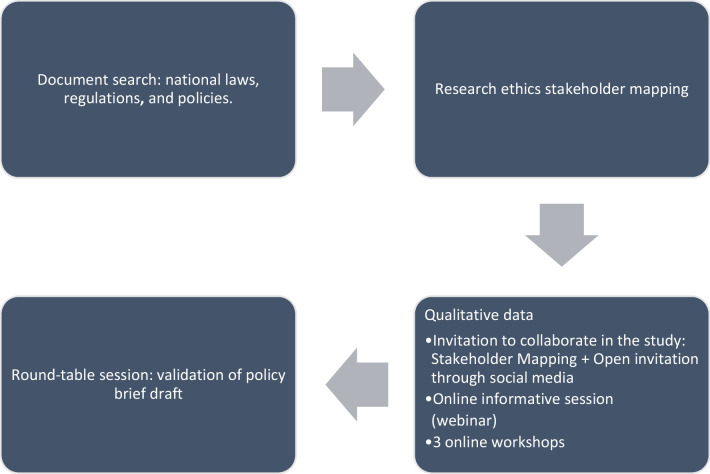
The study’s model and exploratory approach, GoEtiCA study, 2021

An exploratory approach was followed, triangulating data collected with multiple tools collated at different points in time (Fig. 1). The CA-DR region comprises eight countries; ethical approval for the study was obtained from six of them (Dominican Republic, Honduras, Costa Rica, Panama, Guatemala and El Salvador). We were unable to contact research ethics experts from Belize and Nicaragua; therefore, local ethical approval was not sought, and these two countries were not included in the study.

### Document search: national laws, regulations and policies

A search was conducted for all available regulatory documentation within participating countries, including laws, regulations and policies specific to research ethics. Summary charts were organized for each country; charts were reviewed for accuracy, and information on each country’s ethical governance and regulations was updated as necessary.

### Research ethics and stakeholder mapping

A purposeful heterogeneous sampling strategy was employed [20]. A key stakeholder mapping exercise was conducted to identify researchers, representatives of community-based organizations (CBOs), research ethics committee (REC) members and government officials residing or working in institutions in CA-DR. Contact information for the study’s target population was identified via public and freely accessible public and private organization websites, including ministries of health (MoHs) and other ministries and institutions associated with higher education, scientific and technological endeavours, national commissions on bioethics (NCBs), health research centres and institutes, RECs and CBOs within the health sector. REC members were identified through the Central America and Caribbean Network of Research Ethics Committees [21]. The mapping exercise resulted in a database containing 356 key stakeholder contacts from the eight countries of the Central American Integration System (SICA, by its acronym in Spanish).

### Qualitative data: workshops

**Fig. 2:**
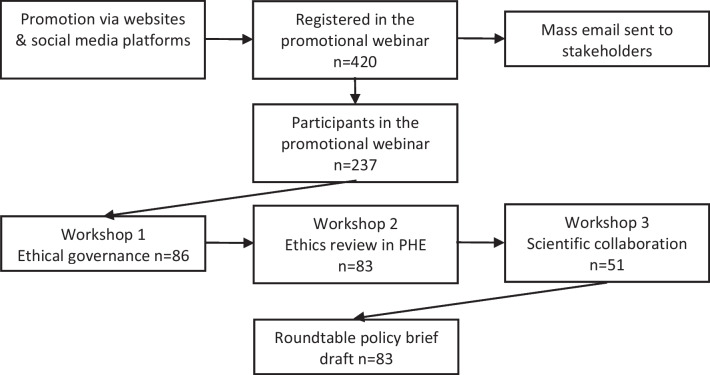
Number of participants by activity, GoEtiCA study, 2021

Participants from six countries (Costa Rica, El Salvador, Guatemala, Honduras, Panama and the Dominican Republic) were invited by email to an online informative session. An open call was also published on social media^1^ inviting researchers across the region to the online session. Participants completed registration voluntarily, with documented informed consent. During the session, objectives and methods of the study were presented and attendees were invited to participate in study activities. Figure 2 details the number of participants by activity. Data collection was completed by 23 April 2021.

**Fig. 3:**
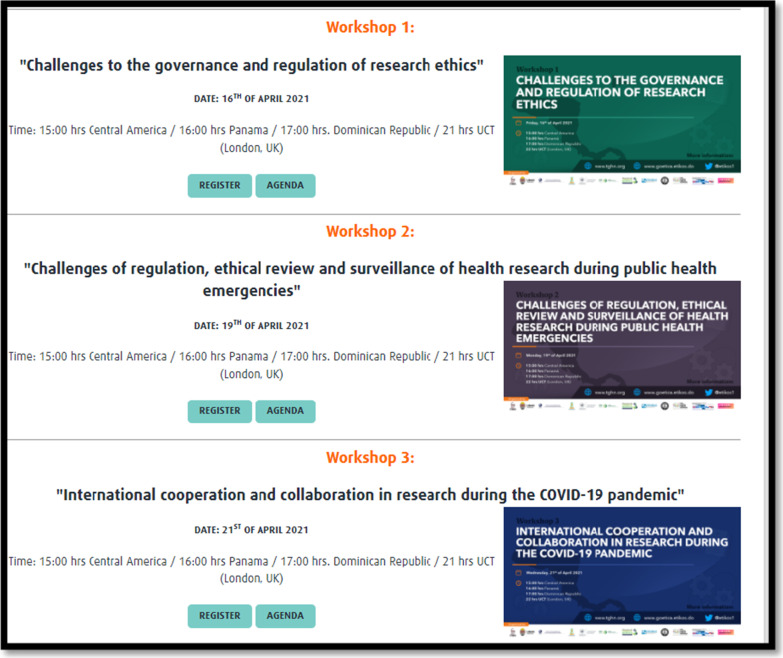
Thematic clusters as shown on promotional banners used for announcing the workshops, GoEtiCA Study, 2021

Three workshops were organized, each focusing on a thematic cluster, as detailed in Fig. 3.

Participants from the stakeholder mapping exercise and webinar attendees were invited to the workshops. Key research stakeholders from Latin America (Mexico, Colombia, Venezuela and Argentina) who attended the online informative session as subject experts were asked to participate in the workshops as listeners-observers. Each workshop was conducted and recorded using the Zoom platform meeting function and lasted 2.5 hours. Participants were informed that their involvement in the study was voluntary, and consent was obtained at the time of individual voluntary registration for workshops.

**Fig. 4:**
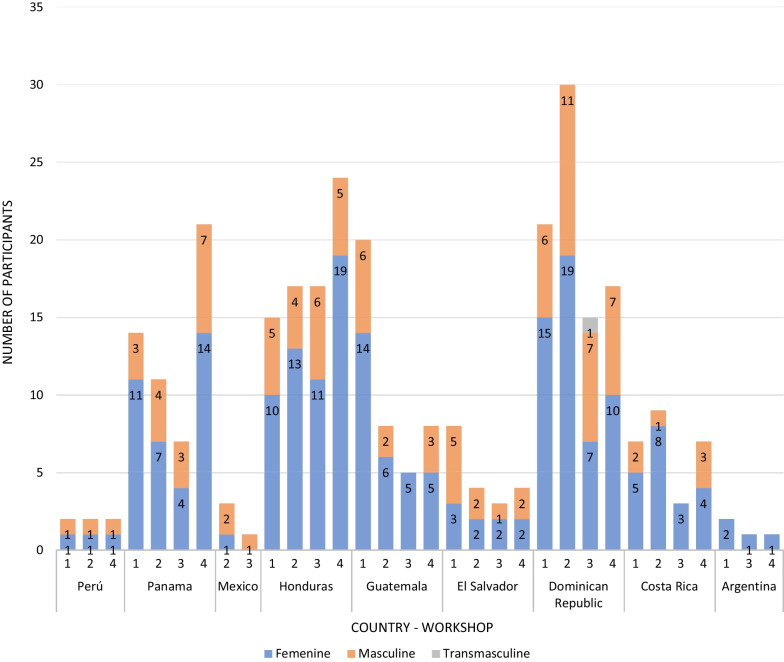
Participants in each of the study’s four events, by country and gender, GoEtiCA study, 2021. *Legend* 1 = workshop 1; 2 = workshop 2; 3 = workshop 3; 4 = roundtable

Figure 4 shows participant attendance at each of the four participative events (workshops 1–3, roundtable) by country.

**Fig. 5:**
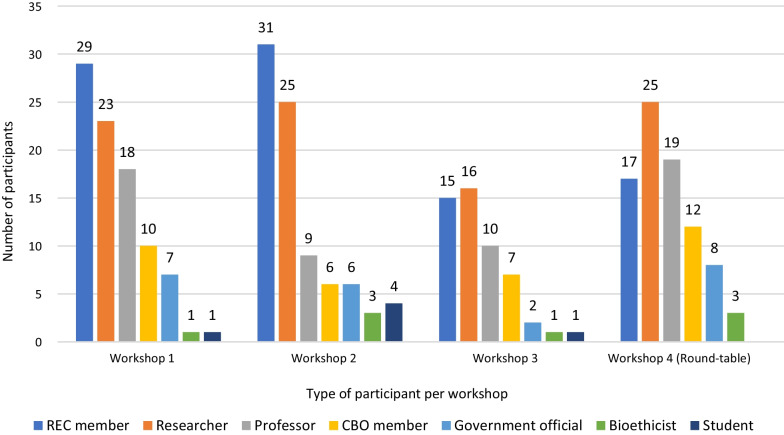
Workshop and roundtable participants by research stakeholder group, GoEtiCA study, 2021. *CBO* community-based organization, *REC* research ethics committee

Figure 5 shows workshop and roundtable participant distribution by research stakeholder group. Findings were summarized by thematic cluster.

During each workshop, presentations were offered on the thematic cluster, triggering questions posed by research team members (Table 1). Workshop attendees were encouraged to participate freely. The audience was divided into groups using the Zoom breakout rooms function, and subthemes were discussed. Breakout rooms were moderated by a research team member; a research assistant took notes during the discussion. The moderator used a discussion guide prepared beforehand as a guideline to channel the dialogue towards the specific objectives set forth for this study. Participants were warned against confidentiality breaches; they were asked not to disclose any information revealed in the breakout rooms.

**Table 1:** Sample workshop discussion questions by thematic cluster

Guide questions used during workshops
THEMATIC BLOCK 1. Governance and regulation of research ethics
1. According to your experience, whether in your country or Central America in general, have ethical standards for human research been adopted following international guidelines?
2. Does the level of regulation (law, decree, resolutions, rules, regulations, guidelines, national guidelines and standard operating procedures) include recommendations made by international ethical regulations?
3. What are some possible paths to advance an agenda that strengthens the governance and regulation of health research in your country/region?
THEMATIC BLOCK 2. Preparedness and coordinated research response to the COVID-19 pandemic
1. Regarding the COVID-19 pandemic, how do you assess ethics review preparedness and response at the national and regional levels?
2. Could the articulation of collaborative efforts during the pandemic be described as successful (e.g. efficient, effective)?
3. In the context of the COVID-19 pandemic, what has been the response to the ethical review of protocols related to COVID-19? Could you briefly describe your experience?
4. What are the challenges to move forward in the context of COVID-19 and strengthen the capacity for ethical review during public health emergencies?
THEMATIC BLOCK 3. Collaboration, international cooperation and community participation
1. Does the current regulatory framework for health research stimulate research and promote collaboration? Does it encourage intersectoral, interdisciplinary, inter-institutional collaboration?
2. Do prevailing practices in your country promote open dialogue and protection for participants in scientific research among researchers, regulators and the public?
3. From your point of view, what kind of international cooperation is required in the Central American and Caribbean region?
4. Have community organizations representing populations or individuals taken part in the formulation of research policies and ethical standards? If so, to what extent?
5. How could communities be involved in these governance processes?

Breakout room discussions were followed by a plenary session, where each group presented their conclusions.

A pragmatic thematic analysis followed. The summarized rapporteur text from breakout room activities, notes from research assistants and moderators, and records of participants as well as chat interactions during the workshops were sources of qualitative data. To ensure confidentiality, all data were stored securely on encrypted drives, password-protected through secure login; use was restricted to the study team and authorized personnel.

### Roundtable session: validation of policy brief

A fourth workshop (roundtable) was organized to present preliminary research results. Six panellists representing each of the countries involved in the study offered a succinct commentary, focused on strengthening research ethics governance in the region. Specifically, emphasis was placed on building consensus around a proposal for regional ethical governance policy and regulation of health research in the CA-DR region in response to the COVID-19 pandemic and in preparation for future PHEs.

## Results

### Theme 1: governance and regulations for research ethics

#### The status of research ethics systems and adoption of international ethical standards for human research

In our document search, we found that health research regulation across the CA-DR region is very uneven, ranging from no regulation at all to the application of highly restrictive measures to research. Only Costa Rica and Panama have laws regulating health research. The MoHs in Honduras, Guatemala, El Salvador and the Dominican Republic have mandates that regulate health research; however, regulation is not fully established and may sometimes be lax, unwritten and/or informal, with incipient research ethics systems.

Only three countries (50%) have established a formal written policy for health research and formal research priorities. However, all six countries included in this study have official agencies in charge of regulating health research activities. Except for Honduras, each country has a national REC (NREC) in charge of ethics review or, as in Costa Rica, in charge of supervising local committees that carry out ethics review. Only in Costa Rica and Panama are the NRECs mandated by law. The NRECs in El Salvador, the Dominican Republic and Guatemala operate under the mandate of the MoH.

For example, when asked about research ethics policies and regulations, one study participant described the Honduran system as fragmented or incomplete:

*I think that we lack that regulatory framework. [The system] is perceived as unfinished, sometimes fragmented, stagnated. There is still no vision of that regulatory framework…We are in the very early stages.* (Professor/researcher, Honduras, Workshop 1)

Only Panama and Costa Rica have established a system for registration and accreditation of RECs. National training programmes in research ethics are notably absent, even for members of the NREC (Table 2).

**Table 2:** Ethical governance and regulations within the NHRS

NHRS and NRES indicators	Costa Rica	El Salvador	Guatemala	Honduras	Panama	Dominican Republic
1. Law on HR	Biomedical Research Regulatory Law No. 9234	No	No	No	Law 84 of 19 May 2019 that regulates and promotes research for health and establishes its stewardship and governance Law 56 of 14 December 2007, which creates the National Research System	No
2. National policy for HR	National Health Plan 2016–2020	National Health Research Policy	No	No	National Health Policy and Strategic Guidelines 2016–2025	No
3. HR priorities	No	National Health Research Priorities Agenda, 2018–2024	Research Areas and Priorities for Health in Guatemala 2014–2019	Research Agenda for Health, Honduras 2015–2020	National Agenda of Research Priorities for Health (ANPIS) Panama 2016–2025	No
4. Decrees, codes, administrative resolutions on HR	Decrees No. 39061-S dated 8 May 2015 and No. 39533-S dated 11 January 2016 Regulation of Law No. 9234 Decree No. 41183-S of 11 May 2018 (Regulation of adult stem cell research) Regulations to the Biomedical Research Regulatory Law—Executive Decrees 39061-S and 39533-S Regulations for the operation of the National Health Research Council (2018)	Inter-institutional cooperation agreement between the Ministry of Health through the National Institute of Health, the National Committee on Health Research Ethics and the National Directorate of Medicines Operating Manual of the National Committee on Health Research Ethics	Ministerial Agreement No. 206-2021	Decree Number 65-91 (Issued on 28/05/1991) Health Code (Gazette No. 26509 of 06/08/1991) Articles 175 and 176 Health Code, research provisions in Articles 175 and 176 (Decree No. 65-91) Provisions for the Regulation and Sanitary Control of Clinical Trials on Human Subjects	Executive Order No. 179 of 8 June 2018 regulating research with tissues and cells of human origin Regulations of the National Research Bioethics Committee	Administrative Resolution No. 013625 of 2 August 2000. Ministry of Public Health
5. Governmental agency responsible for research ethics	National Health Research Council (CONIS)	National Health Research Ethics Committee	National Health Ethics Committee	Ministry of Health, General Directorate for the Surveillance of the Regulatory Framework	National Committee on Research Bioethics, Ministry of Health	National Council on Bioethics in Health (CONABIOS)
6. Budget allocated to sustain the research ethics regulatory work	Yes	Yes	Yes	No	Yes	Yes
7. Scope of the regulation for RECs	Registry, creation and accreditation	Registry and creation	Registry and creation	Registry and creation	Registry, creation and accreditation	Registry
8. National mandate to promote research ethics training	Yes	No	No	No	Yes	No
9. Existence of an accredited national research ethics training programme	No	No	No	No	Yes	No
10. Training programme available for new members of the NREC	No	No	No	No	Yes	No

All websites and documents are in Spanish

*HR* health research; (*N*)*REC* (national) research ethics committee; (*N*)*HRS* (national) health research systems; *NRES* national research ethics systems

### Theme 2: ethics review preparedness and response to PHEs: COVID-19 times

#### Ethics review preparedness and response at the national and regional levels

Formal national research priorities specific to COVID-19 were not identified in any of these countries (Table 3). In spite of lack of formalization of priorities and the identification of funding, study participants reported an increased number of protocols and the inability to respond to the demand.

*There has been lack of coordination, which was exacerbated during the pandemic with the increase in demand for COVID-19-related reviews.* (REC member, Dominican Republic, Workshop 2)

During the COVID-19 pandemic, five out of six countries adopted changes to the ethics review process implemented due to the need to meet virtually and adopt electronic submission of protocols (Table 3). Only Panama officially established an accelerated procedures for ethics review.

**Table 3:** Ethics review preparedness and responses, by country, GoEtiCA study, 2021

	Costa Rica	El Salvador	Guatemala	Honduras	Panama	Dominican Republic
1. Health research priorities identified for COVID-19	No	No	No	No	No	No
2. Ethics review process adopted-adapted for COVID-19^a^	Yes, the guideline “Recommendations for carrying out biomedical research during the health emergency for COVID-19 in Costa Rica”, includes ethical review guidelines	Yes, National Commission on Research Ethics minutes, establishing recommendations in dialogues on research ethics during pandemics	Yes, Evaluation criteria for outbreak or health emergency-related research	No	Yes, Resolution No. 373 of April 2020, accelerated procedure for a PHE. Resolution 512, 19/06	Yes, Communication, CONABIOS in times of COVID-19
3. Number of COVID-19 studies registered in ClinicalTrials.gov by 08/2021	4	15	2	2	3	4
4. Number of studies on COVID-19 approved by the NREC by 08/2021	21	14	19	NA	89	13
5. Registry of protocols approved	Yes	Yes	Yes	No	Yes	Yes

*NA* not applicable, does not have a NREC; *NREC* national research ethics commission

^a^Document(s) indicating the adaptation or modification of the ethics review process to respond to the COVID-19 pandemic

Ethics committee members have acknowledged that they do not have expertise in the use of technology and digital tools for ethics review. They have been reluctant to incorporate virtual work, due to factors such as age, accessibility, interest, time, duplication of effort and work overload.

*Virtuality and the use of technology for reviews have their limitations. Some members did not adapt to it, some withdrew since they could not handle it, and others continued to collaborate, but by email.* (REC member, El Salvador, Workshop 2)

Double review processes with ethics review by more than one REC caused duplications that increased the burden for both ethics committee members and researchers (e.g. protocols submitted to several committees at the same time in some countries).

#### Articulation of ethics review collaborative efforts during the pandemic

There is a gap between the number of COVID-19 studies registered in ClinicalTrials.gov by August 2021 and the number of studies on COVID-19 approved and reported by national research ethics authorities (Table 3). Five out of six countries have a registry of approved protocols, but none of them requires prospective registration of clinical trials. We could not find evidence of collaborative efforts on ethics review during the pandemic, for example, to avoid double review processes or reduce the number of reviews by various ethics committees.

Table 3 presents a summary of the ethics review preparedness and responses to PHE during the time of COVID-19.

### Theme 3: collaboration and international cooperation during the COVID-19 pandemic

#### Regulatory framework for health research and promotion of collaboration (intersectoral, interdisciplinary, inter-institutional)

Formal international cooperation between health research institutions was found to be almost nonexistent within the region. Participants in the workshops were unable to contribute substantially to this question. We could not identify studies undertaken by at least two collaborators from the LAC (Latin America and the Caribbean) region.

#### Community engagement in research ethics governance processes

The need for community participation to establish public policies on research ethics was identified by study participants. An example of best practice of good governance was found in Panama (Table 4), where a regulatory framework was developed with the strong participation of civil society.

*Three years ago, we were working on the regulatory framework of our country; enforcement began in force in 2019 and it is now Law 84. It was an important exercise for all stakeholders involved in health. It was a win-win for all actors: research subjects, researchers, and the country.* (Government official, Panama, Workshop 1)

None of the other countries indicated having involved the community in the development of research ethics regulations.

**Table 4:** Guidelines for community engagement, responsible conduct of research and coordination mechanisms between RECs, reported by country, GoEtiCA study, 2021

	Costa Rica	El Salvador	Guatemala	Honduras	Panama	Dominican Republic
1. National guidelines for community engagement in research	No	No	No	No	Yes	No
2. Community involvement in the establishment of policies and/or priorities for health research	No	No	No	No	Yes	No
3. National guidelines for conducting research responsibly	No	No	No	No	Yes	No
4. Number of RECs	18^a^	23^a^	10	11	13^a^	20^b^
5. Coordination mechanism between RECs	Yes	Yes	No	Yes	Yes	No

^a^Accredited; ^b^registered

#### Prevailing practices in countries to promote open dialogue and protection for participants in scientific research among researchers, regulators and the public

Other important issues are the promotion of scientific collaboration among stakeholders and the promotion of responsible conduct of research. There is little awareness of the importance of responsible conduct in research. Another participant stated:

*Responsible behaviour implies that researchers must become aware of the importance of adhering to responsible practices in the development of research. This awareness can be strengthened through our work context and from training of human resources, beginning at the undergraduate level. At the postgraduate level, there must be more training.* (Professor, Honduras, Workshop 3)

Table 4 presents a summary of the international cooperation and collaboration among research stakeholders described by study participants from each country.

#### Challenges to move forward in the context of COVID-19 and strengthen governance and regulation of health research

The main challenge identified was increasing the capacity to respond effectively to ethical review requests during PHEs. Most opinions were directed towards the formalization of the role of ethics committee members and the need for training opportunities for all involved in the research process: researchers, REC members, and undergraduate and graduate students.

(i) *Formalization of the work of RECs* Many ethics committees in the region lack resources to carry out their functions. Financial and technological resources are missing primarily for equipment and support staff, as well as REC member compensation for review and oversight of research protocols. Despite efforts to adapt the review process, the ad honorem status of most ethics committee members in the region was identified as one of the reasons for delays in the review and approval of research studies. Therefore, REC needs should be considered in institutional budgets to cover the costs of training and time for protocol review. Time and effort of members within institutions should be recognized as part of their job responsibility, and compensation for external members should be provided.
(ii) *Capacity-building in research ethics and collaborative practices* Recommendations were oriented towards the development of training programmes in research ethics, such as workshops with active learning methods, as well as international exchange programmes and training programmes for undergraduate and graduate students, including master’s degrees in research ethics. It was highlighted that scholarships to ensure access to training are needed. All this could be accomplished through international and regional collaboration.

## Discussion

The CA-DR region has significant potential for developing strong and solid mechanisms for sharing experiences to improve research ethics governance. This potential is evident in examples of progress across the different countries in the region in terms of regulatory frameworks, prioritization of initiatives, funding, ethics committee networks, ethics committee training and community representation in research [16, 22–24]. The GoEtiCA participatory action research project allowed a broad audience to meet virtually and provide insight, experiences and recommendations. We acknowledge differences in the level of knowledge in the composition of our participant sample and stakeholders; however, democracy is all about accepting different opinions. Our findings align with the conclusion of Rodriguez and Lolas [25]: “Research integrity only will become alive with public debate and reflection about scientific advances, while preserving human dignity and autonomy. Research ethics should be discussed with the general public and integrate their views in the process of policy development. This was the main gain of the collaborative effort.”

### Challenges to the governance and regulation of research ethics

Many countries were shown to be insufficiently prepared to respond to the pandemic, as their own research ethics systems were not opportunely and fully developed. While pragmatic solutions are needed in times of crisis, such as the COVID-19 pandemic, time and resources must be invested beforehand by host countries, in particular to develop NRES as part of NHRS. As Mathur [26] stated, “An ethically conscious, well informed and updated governance framework which identifies the relevant stakeholders, defines their roles and responsibilities, lays down an implementation plan and a monitoring strategy, can safeguard the ethical values of the society, promote good science and deliver better outcomes.” Strict regulations are not always best; an appropriate balance is needed between regulating and promoting research both during and before/after a pandemic. Collaboration within and across regions is key to overcoming obstacles and working towards robust research ethics governance at national and regional levels [27].

Aguilera et al. [27] found that “Most countries have adopted legal instruments to govern research with human participants and have implemented national bodies tasked with the oversight of RECs. However, performance regarding ethics training policies and clinical trial registration was less advanced, and efforts to adopt policies on responsible conduct of research and accelerated ethics review of emergency research did not meet the PAHO objectives in most countries.” Countries must do more to develop policies, procedures and standard operating procedures for fast-tracked and rigorous ethics review during emergencies [27].

### Challenges to ethics review during COVID-19 and future PHEs

Despite the existence of the Council for International Organizations of Medical Sciences (CIOMS) Ethical Guideline 20 on Research in Disaster and Disease Outbreak Situations [18], implementation of operating procedures based on this guideline has been limited. Palmero et al. [22] concluded that “[c]ontinuing efforts should be directed to strengthen [Latin American] countries’ research capacity to respond timely and ethically to future health emergencies”. In this study we confirm that there is a clear need to improve ethics review practices in light of the difficulties identified by study participants.

### Challenges for international collaboration and cooperation during the COVID-19 pandemic

Articulation and coordination of the research response to the pandemic in CA-DR was weak. The number of COVID-19 studies registered in ClinicalTrials.gov by August 2021 showed the low intensity of the research environment in the region and could indicate that studies are not registered prospectively in clinical trial registration platforms, although most of the research ethics national bodies internally do register the approved protocols.

In the CA-DR region, accredited training opportunities are scarce. As training programmes are not always available, the most common sources of training are international programmes that are limited to conceptual aspects of training. Most often, these programmes do not provide specific details on national regulations and practices. International courses cannot substitute national training programmes that are comprehensive, accredited and adapted to local settings. One option to optimize resources may be training programmes with a regional perspective. Ángeles-Llerenas et al. [28] concluded that “[i]nvestments in REC member training and infrastructure are needed to ensure compliance of REC evaluations with the standards for ethical conduct of research”.

The uniqueness of this work lies in its collaborative and participatory methodology, where diverse stakeholders in health research systems in different countries were engaged and involved in the study. It was essential to start by motivating stakeholder participation, promoting dialogue and improving communication and coordination. This preparation allowed moving towards consensus-building around identified goals, which were the allocation of resources for priority research areas and the strengthening of ethical governance and regulations in health research.

A policy brief proposal was prepared from this deliberative and open dialogue (Additional file 1), representing the perspective of a broad range of research stakeholders in the region. GoEtiCA researchers presented the policy proposal to the Council of Ministers of Health within SICA (COMISCA).

### Limitations

Ethics approvals of the GoEtiCA protocol in participating countries took longer than planned, primarily because the process began at the end of 2020, when several ethics committees in the region were experiencing an end-of-the-year recess. The GoEtiCA protocol also underwent substantial changes during the ethics review process and was updated to include requirements from the different ethics committees. These updates created additional delays that were not considered in the study’s initial planning phase. These GoEtiCA protocol issues are clear indicators of the challenges investigators face when conducting multisite and multi-institutional research studies. In an ideal scenario, all the RECs could have come together for a single, concurrent REC review that would work for all involved.

The other major limitation of the study was the inability to recruit participants from all Central American countries. To obtain ethical approval, the research team made all possible efforts to identify in-country collaborators, efforts that included dissemination and local promotion of the study to key players and searches for comprehensive information on local regulatory documents and practices in all Central American countries. Initially, the study aimed to include all the countries within the SICA region, but that was not possible since a local research collaborator could not be identified or effectively contacted in Belize and Nicaragua. Notwithstanding the difficulties in conducting this study, it is important to highlight the transparency, strong collaboration and participatory approach that led to notable results.

## Conclusion

Frameworks and strategies to improve research ethics governance within the CA-DR region are needed. Although there are recent initiatives that may have a positive impact in terms of research collaboration and capacity-building in the region, this study found that none of these initiatives is focused on research ethics and its governance [29, 30]. There is a history of previous research advocacy in the region [31] as part of international collaboration strategies, but these strategies and the advocacy are still at the beginning stages. The COVID-19 pandemic has provided an opportunity to plant the seed of collaboration among RECs [32]. However, formalization of collaboration remains necessary; informal, unplanned activities are marginal responses to the COVID-19 pandemic. Challenges experienced in the CA-DR region are not unique to this region. The learning process from other epidemics regarding the role of preparedness seems to be slow [33], as some countries were unable to respond adequately and make the changes demanded by the current pandemic context. Ethnographic studies may be needed to understand cultural practices and regulatory differences in research ethics governance. It is possible to create and establish mutual trust and equitable scientific collaboration that favours rapid accessibility and timely information-sharing; the feasibility of adaptation to the local requirements of each nation can likewise be assessed and determined [34].

## Supplementary Information

**Additional file 1.** Proposal for the governance and ethical regulation of health research in Central America and the Dominican Republic in the context of the COVID-19 pandemic.

## Data Availability

Datasets generated during and/or analyzed during the current study are available from the corresponding author on reasonable request.

## Abbreviations

CA-DR: Central America and the Dominican Republic
CBO: Community-based organization
CIOMS: Council for International Organizations of Medical Sciences
MoH: Ministry of Health
NCB: National commission on bioethics
NHRS: National health research system
NREC: National research ethics committee
NRES: National research ethics system
PHE: Public health emergency
REC: Research ethics committee
SICA: Sistema de la Integración Centroamericana (Integration System of Central America and the Dominican Republic)

## Footnotes

1 The open call was released through social media of partner institutions in the region and the Etikos (https://etikos.do/) and The Global Health Network (https://tghn.org/) webpages.
