# Supplementary material for: Strengthening research ethics governance and regulatory oversight in Central America and the Dominican Republic in response to the COVID-19 pandemic: a qualitative study
Source: Health Res Policy Syst. 2022 Dec 23;20:138. doi: 10.1186/s12961-022-00933-z (PMC9786418; doi:10.1186/s12961-022-00933-z)
Supplement: Supplementary file 1 — Additional file 1. Proposal for the governance and ethical regulation of health research in Central America and the Dominican Republic in the context of the COVID-19 pandemic. [file 12961_2022_933_MOESM1_ESM.docx]

**Strengthening** **research ethics governance and regulatory oversight in Central America and the Dominican Republic in response to the COVID-19 pandemic: a qualitative study**

**Additional File 1**

**POLICY BRIEF**

**Proposal for the governance and ethical regulation of health research in Central America and the Dominican Republic in the context of the COVID-19 pandemic**

1. **Promote the importance of health research as a response to emergency situations, disasters, and disease outbreaks recognizing that preparedness and effective responses in terms of research ethics are required.**
   1. Learn from countries in the region that have a robust regulatory base. It is recommended to seek mechanisms for harmonization of research ethics regulations in the region.
   2. Governments in each country should recognize ethics as a central part of the research process to increase quality of research in the region and, above all, to effectively protect people and populations that participate in human research.
   3. Increase quality of studies conducted in the region to be more competitive and expand fundraising for relevant research to solve the region's health problems.
   4. Promote financing for health research in emergency situations and disease outbreaks, with clearly established norms and due ethical rigor for research, allowing expediting processes to benefit an effective and timely response to health problems.
2. **Implement application of ethical principles of research as a key element to safeguard human dignity and the rights of participants and communities in scientific research.**
   1. Adequately fund the ethics review system and, directly, research ethics committees.
   2. Promote ethical research and responsible conduct aligned with internationally recognized standards.
   3. Achieve automation of protocol registration and monitoring processes by ethics committees to optimize review and response times, especially in contexts of health emergencies such as the COVID-19 pandemic.
   4. Actively incorporate community representatives in research ethics committees and research projects.
   5. Implement a Regional Master's Degree Program for members of bioethics committees and international courses in research methodology to strengthen scientific collaboration.
3. **Adequate operational procedures for ethics review in response to emergency situations and disease outbreaks**
   1. Establish mechanisms for implementation, follow-up, monitoring and evaluation of the strategic plan on research ethics, with emphasis on emergencies, disasters, and epidemics.
   2. Ensure that countries establish a minimum budget dedicated to health research ethics and health research in emergency situations. Governments should be prepared for these emerging situations.
4. **Strengthen mechanisms for citizen participation, communication between community members, researchers, ethics committees and health authorities through deliberative spaces.**
   1. Regulations should be created in a participatory and open manner and be reviewed and updated periodically, given the dizzying changes and challenges faced by nations and regions to maintain collective health.
5. **Increase the level of scientific collaboration and international cooperation in research ethics.**
   1. Create or strengthen a Central American and Dominican Republic network for the development and follow-up of governance and ethical regulation of research, which can increase collaboration in the region.
   2. Train and achieve the formation of a critical mass of research methodologists in each country of the Central American and the Dominican Republic Region to strengthen scientific collaboration and quality ethical review and increase the uptake of multinational health research opportunities.
